# Effects of Oral Exposure Duration and Gastric Energy Content on Appetite Ratings and Energy Intake in Lean Men

**DOI:** 10.3390/nu8020064

**Published:** 2016-01-26

**Authors:** Anne G. M. Wijlens, Cees de Graaf, Alfrun Erkner, Monica Mars

**Affiliations:** 1 Division of Human Nutrition, Wageningen University, 6700 EV Wageningen, The Netherlands; anne.wijlens@wur.nl (A.G.M.W.); kees.degraaf@wur.nl (C.G.); 2 Nestlé Research Center, Nestec Limited, PO Box 44, 1000 Lausanne 26; Switzerland; alfrun.erkner@rdls.nestle.com

**Keywords:** satiety, appetite, energy intake, oral exposure, gastric energy content

## Abstract

Studies show that longer oral exposure to food leads to earlier satiation and lowers energy intake. Moreover, higher energy content of food has been shown to lead to higher satiety. Up to now, it has not been studied systematically how oral exposure duration and gastric energy content interact in satiety regulation. Thirty-seven men (22 ± 4 years, 22 ± 2 kg/m^2^) participated in a randomized cross-over trial, in which we independently manipulated: (1) oral exposure duration by modified sham feeding (MSF) for 1 or 8 min; and (2) energy content of gastric load (GL) by a nasogastric tube: 100 kcal/500 mL or 700 kcal/500 mL. Outcome measures were appetite ratings and subsequent energy intake from an *ad libitum* meal. Energy intake was 35% lower after the GLs with 700 kcal than with 100kcal (*p* < 0.0001). All appetite ratings were lower in the 700 kcal than in the 100 kcal treatments (area under the curve (AUC); *p*-values ≤ 0.002); fullness was higher and prospective consumption was lower in the 8 min than in the 1 min MSF treatments (AUC; *p*-values ≤ 0.02). In conclusion, the current showed that a GL of 700 kcal/500 mL *vs.* 100 kcal/500 mL increased satiety and lowered energy intake. No additional effects of oral exposure duration could be observed, presumably due to the high contrast in energy between the manipulations. Future research should also focus on the role of oral exposure as such and not only the duration.

## 1. Introduction

Food intake is an episodic activity, that is, people eat in short time-moments with intervals of no eating during which people feel satiated and hunger feelings are suppressed [1]. A cascade of different psychological and physiological processes is elicited during and after food consumption [1]. Stomach filling and presence of nutrients in the duodenum have shown to affect meal size [2,3,4]. Important factors in these early digestive processes are the volume, nutrient composition, and energy density of the foods in the stomach.

For satiation—the process that brings people to end their meal—it has been shown that oro-sensory properties of a food, such as the palatability, structure, and taste, are key players. The last decade it has become clear that that especially the time during which the food is present in the oral cavity has an important role in satiation [5]. For example prolonging oral exposure by altering the physico-chemical structure of a food [6,7,8], lowering the rate of eating [9], for example by changing bite or sip size [10,11,12,13], and mode of consumption [14,15], have shown to lead to earlier satiation. 

Cecil and colleagues have hypothesized, based on a series of studies, that oro-sensory signals are especially important for the magnitude of physiological feed-back in order to have optimal satiation and digestion [2]. This has been illustrated by studies in which food was infused directly in the stomach by a nasogastric-tube. For example, Cecil has shown that consumption of soup resulted in earlier satiation compared to intra-gastric infusion of the same soup [16]. A recent study of Spetter *et al.* showed that intra-gastric infusion of chocolate custard lead to delayed and higher responses in insulin and GLP-1 compared to normal consumption [17]. These observations suggest that oro-sensory signals are important in preparing the body for optimal digestion.

However, the effect of oro-sensory exposure in combination with gastric filling on food intake is barely investigated. Cecil *et al.* [16] investigated this indirectly; they observed differences in energy compensation after ingestion of carbohydrates compared to fat when ingested orally, whereas in their second experiment, no such effects were found after intra-gastric infusion [16]—suggesting that oral exposure, in other words identification of the food, is essential for optimal energy compensation. Previously, we performed a study in which we investigated the effect of oral exposure duration—by means of a modified sham feeding paradigm (MSF)—and different iso-caloric volumes of gastric filling on energy intake. We found that with longer oral exposure duration (8 min MSF) subjects ate on average 17% less energy in their subsequent meal, whereas in the short conditions (1 min MSF) non-statistically significant reductions were found [18]. This shows that oro-sensory exposure duration can affect subsequent satiety—the suppression of hunger and the urge to eat in the time after consumption. 

Recently, we conducted a study with a similar protocol in which we again manipulated oral exposure duration by MSF—1 or 8 min—in combination with isovolumetric loads with different energy content—100 or 700 kcal—by means of intra-gastric infusion [19]. We observed a clear inhibition of gastric emptying after the high energy load, but oral exposure duration did not modify this effect. Despite that, we did not see an effect of oro-exposure duration on gastric emptying in our previous study, we cannot rule out that other physiological factors, like satiety related peptides, might affect food intake after 30 min. In the previous study, we did not have the opportunity to include a test-meal, as this would have affected our primary outcome, gastric emptying. We, therefore, conducted the current study. We now hypothesized that a longer oral exposure duration combined with a high energy gastric load (GL) would lower energy intake at a subsequent meal. In addition, we expected satiety ratings to be higher after the longer oral exposure duration and the high energy GL.

## 2. Methods

### 2.1. Subjects

Healthy young men were recruited from Wageningen and surroundings. Men had to be between 18 and 40 years old with a normal BMI (≥18.5 to ≤ 25.0 kg/m^2^). They needed to have a good—self-reported- mental and physical health and a stable body weight (defined as: a maximum body weight change in the last 2 months of 5 kg). Exclusion criteria were: restrained eating behavior (score ≥ 2.26, as determined using the Dutch Eating Beahviour Questionnaire (DEBQ [20]), smoking, gastrointestinal diseases, diabetes, thyroid diseases or any other endocrine disorders, lack of appetite, hypersensitivity or food allergy for products used in the study, or medication or drug use (mild pain relievers available over the counter, such as paracetamol, were allowed).

**Figure 1:**
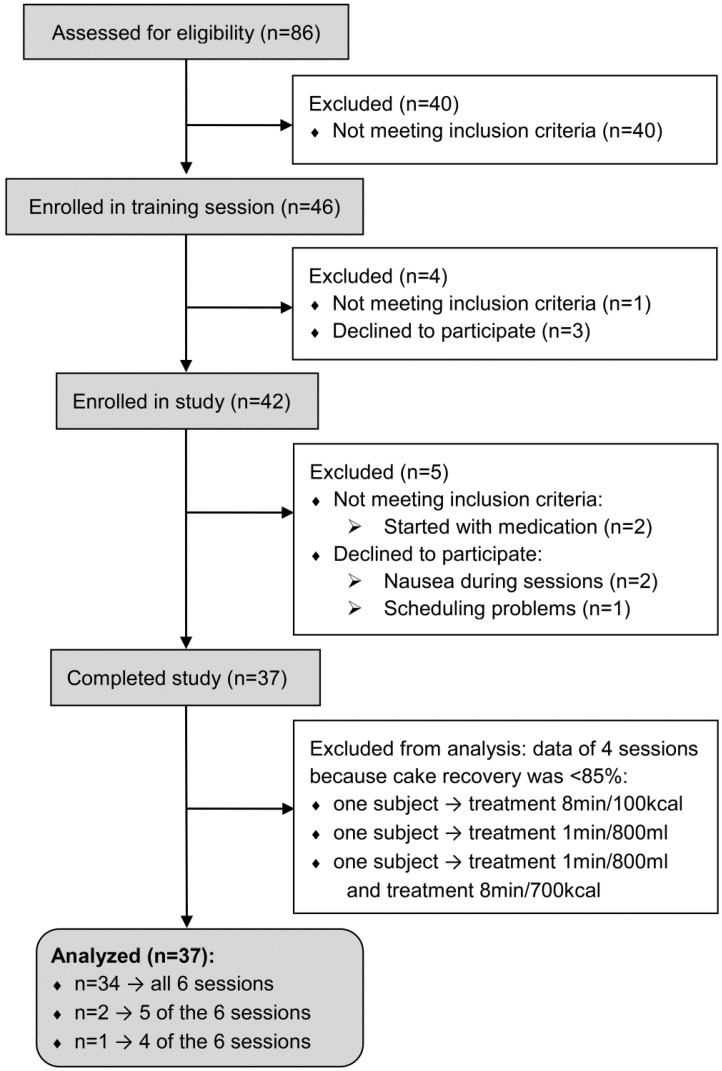
Flow diagram of participants.

In total, 86 men completed the screening questionnaire, of which 56 seemed eligible and were invited for an additional screening and information meeting (Figure 1). The majority of interested males were studying at Wageningen University. Forty-six of them met all inclusion criteria. These men then had to successfully complete a training session with all study procedures of treatment 8 min/700 kcal (Table 1) to be further enrolled in the study. Three men stopped after the training session because they found the tube intubation too invasive, and one man was excluded because he had a recovery rate of 83% during MSF (see Section 2.4.1). The remaining 42 men were enrolled in the study. During the study, five men withdrew: one because of scheduling problems, two because of nausea during the sessions, and two because they started medication. The study was performed from May until December 2011.

**Table 1:** Outline of the six conditions.

	Duration MSF (min)	Recovery MSF (%) ^c^	Energy Content GL (kcal)	Volume GL (mL)	Infusion Rate GL (mL/min)
Control ^a^	*-*	*-*	*-*	*-*	*-*
1 min/100 kcal	1	95 ± 4	100	500	62.5
8 min/100 kcal	8	97 ± 3	100	500	62.5
1 min/700 kcal	1	94 ± 4	700	500	62.5
8 min/700 kcal	8	98 ± 3	700	500	62.5
1 min/800 mL ^b^	1	95 ± 4	100	800	100

MSF, Modified Sham Feeding; GL, Gastric Load; ^a^ Control condition: subjects were inserted with a nasogastric tube, but they were not orally exposed to food and received no GL; ^b^ This treatment is not discussed in this paper (see Section 2.2); ^c^ Mean (±SEM) recovery rate (%) of cake after MSF, based on dry mass analyses.

In total 37 subjects completed the study. They were on average (mean ± SD) 22 ± 4 years, had a BMI of 22 ± 2 kg/m^2^, and had a restrained eating score of 1.5 ± 0.3 (measured by the DEBQ; [20]). Subjects were unaware of the primary study aim to avoid influence of cognitive effects on energy intake. They were informed that the aim of the study was to examine whether both infusing food via a naso gastric tube simultaneously with chewing food would affect appetite feelings. In total, three out of the 37 subjects who completed the study, were not compliant to the MSF protocol; that is their recovery of cake was lower than 85% (Figure 1, Section 2.4.1). For one subject this was the case for two treatment sessions (treatment 8 min/700 kcal and 1 min/800 mL), for two other subjects this was the case for one treatment session only (treatment 8 min/100 kcal and 1 min/800 mL).

All subjects gave written informed consent before start of the procedures. The study was approved by the Medical Ethical Committee of Wageningen University on 4 April 2011 (NL35319.081.11) and was conducted in accordance with the Declaration of Helsinki. The study was registered in the Dutch trial register at www.trialregister.nl before start of the study (NTR number NTR2779). In- and exclusion criteria were monitored by TFS Develop (TFS Trial Form Support BV, Berghem, The Netherlands). Subjects participating in the training sessions and onwards received financial compensation.

### 2.2. Design

The study had a randomized crossover design with six conditions: five treatments and a control condition (Table 1) with a minimum of five days wash out between conditions. In all six conditions a nasogastric tube was inserted. In the treatment conditions, subjects were orally exposed to food by MSF for 1 or 8 min. Simultaneously with MSF, subjects received a GL low or high in energy content via a nasogastric tube: *i.e.*, 100 or 700 kcal. In the control condition only a nasogastric tube was inserted. Additionally, a 1 min/800 mL treatment was included to replicate findings of our previous study [18]; these results are available as supplementary material (Figures S1 and S2).

### 2.3. Randomization

Treatments were randomized by means of 6 × 6 Latin Squares (Williams Design): balanced blocks of six treatment orders were produced with the statistical package SAS (PROC PLAN). Subjects were allocated to a treatment order in order of inclusion. Generation of the Latin Squares, enrolment of subjects and allocation of subjects to the treatment orders was done by the researcher (AW). The study was performed at two locations*, i.e.*, Wageningen University (Wageningen, The Netherlands), and hospital “Ziekenhuis Gelderse Vallei” (Ede, The Netherlands). At both locations the study was carried out by the same researchers.

### 2.4. Study Procedures

Subjects came to the laboratory on six occasions. Arrival time varied between 8:00 AM and 10:30 AM between subjects, but individual subjects were always assigned the same arrival time for their treatment days. Subjects were instructed to avoid any high-intensity exercise from the preceding evening onwards and not to eat anything or drink any energy-containing beverages. Subjects ate a small standardized breakfast at home one hour before arrival at the study centre (Table 2). Subjects were allowed to drink non-caloric drinks before the standardized breakfast; subjects were instructed to keep this as similar as possible on the research days. After breakfast any consumption was prohibited. Compliance to the instructions was monitored with a study diary which was kept by the subjects.

**Figure 2:**
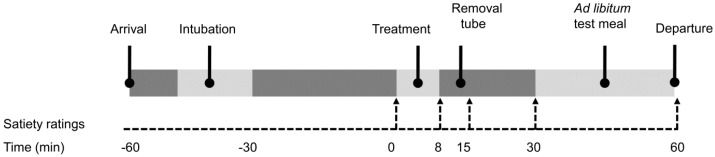
Time-line of the sessions. Subjects arrived at the study centre (*t* = −60), a nasogastric tube was inserted between *t* = −60 and *t* = −30. At *t* = 0 subjects gave satiety ratings for the first time, immediately followed by the treatment (oral exposure and gastric infusion). At *t* = 8 (directly after the treatment), at *t* = 15 and *t* = 30 subjects again gave appetite ratings. Between *t* = 30 and *t* = 60 subjects received a meal *ad libitum* and at *t* = 60 subjects gave appetite ratings once more. The tube was removed between *t* = 8 and *t* = 15.

The time line of the study procedure is shown in Figure 2. A nasogastric tube was inserted within 30 min after arrival. One hour after arrival the treatment started, *i.e.*, subjects started performing MSF and receiving the GL at the same time. The same commercially available sponge cake was used for both MSF and the GL (Table 2).

**Table 2:** Energy content and macronutrient composition ^a^ of the standard breakfast, the sponge cake and the gastric loads.

	Energy (kcal)	Protein (g)	Carbohydrate (g)	Fat (g)	Fiber (g)
Standardized breakfast (per 200 mL) ^b^	198	6.0	26.0	6.0	0.0
Sponge cake (per 100g) ^c^	425	5.9	47.2	23.7	0.7
100 kcal/500 mL gastric load ^d^	100	1.4	11.1	5.6	0.2
700 kcal/500 mL gastric load ^d^	700	9.7	77.7	39.0	1.2

^a^ energy content and macronutrient composition were determined by chemical analysis; ^b^ Friesche Vlag Breaker Aardbei, Arla Foods, Nijkerk, The Netherlands; ^c^ Euroshopper cake, Neerlandia Banket B.V., Bunschoten, The Netherlands; ^d^ Gastric loads consisted of sponge cake mixed with boiled and cooled tap water, water was added up to 500 mL.

#### 2.4.1. Modified Sham Feeding

Subjects were orally exposed to sponge cake with the MSF technique for 1 or 8 min. With this technique subjects are orally exposed by chewing a bite of food without swallowing it and then spit it out at the moment they would normally swallow. After this they take the next bite and repeat the procedure until 1 or 8 min were over. Subjects were free to choose their own bite size and pace of chewing and they received a plate with an excess amount of sponge cake: 100 g for the 1 min MSF treatments, and 350 g for the 8 min MSF treatments. After 1 or 8 min of MSF subjects rinsed their mouth with 25 mL water and spat this also into the cup.

Compliance to the instructions for MSF (not swallowing cake during chewing or after chewing) was checked by analyzing the dry mass of cake that was spat out. We described this method of analysis in a previous study [18]. At the training session the cake recovery after MSF had to be ≥90%. During the treatment sessions recovery had to be ≥85% in order to be compliant to the study protocol. After removing the non-compliant treatments, the mean recovery was 95.8% ± 4.1%, which meant that on average 2.1 ± 3.0 g of the sponge cake was swallowed by the subjects. The recovery percentages differed significantly between treatments (*p* < 0.0001): treatments with 1 min MSF had a lower recovery percentage compared to the treatments with 8 min MSF (Table 1).

#### 2.4.2. Intra-Gastric Infusion

The GLs consisted of the sponge cake (Table 2) blended with water and were administered via a transparent nasogastric tube following the same procedure as described previously [18]. Each day of the study a qualified dietician prepared the required GL treatments according to a strict protocol. The GLs were infused at 37 °C at an infusion rate of 62.5 mL/min. To blind the treatment, the tube was also filled with cake solution during the control treatment. The tube was removed between *t* = 8 and *t* = 15 (Figure 2).

### 2.5. Measurements

#### 2.5.1. Energy Intake

Half an hour after the treatment started (*t* = 30, Figure 2), subjects received an *ad libitum* test meal. To make sure that there was a surplus of food for every subject, the size of the meal was adapted to the estimated energy needs of the subjects. Subjects were divided into three groups based on their daily energy needs (see supplementary Table S1). Energy needs were estimated by multiplying subjects’ basal metabolic rate by their physical activity level [1]. Basal metabolic rate was estimated with subjects’ age, sex, and height [21]. Physical activity level was assessed by a retrospective physical activity questionnaire containing six activities [21,22]. Subjects were then placed in one of the three energy groups by rounding up their estimated daily energy needs.

The test meal consisted of bread rolls with sweet and savory toppings, which are commonly eaten as lunch in the Netherlands (see supplementary Tables S1 and S2). For each topping, an excess amount was served to minimize the effect of portion size on intake. In addition, the bread rolls were served in unusual sizes: *i.e.*, in rolls of 28 g instead of the regular 50 g. Subjects were instructed to eat until they were comfortably full and to stay at the table for 30 min. Furthermore, subjects were instructed to ask for more food if they wanted, however this did not occur. After subjects left, the leftovers were weighed to calculate energy intake.

#### 2.5.2. Satiety and Well-Being

Satiety and well-being feelings were measured right before the treatment started (*t* = 0) and 8, 15, 30, and 60 min after the treatment started (Figure 2). Subjects rated their hunger, fullness, desire to eat, prospective consumption, desire to eat something sweet, desire to eat something savory, thirst, well-being, and nausea on a 100 mm visual analogue scale [1]. The scales were anchored from “not at all” to “extremely”. Satiety and well-being ratings were compared using areas under the curve (AUC). AUCs were calculated from baseline to just before the test meal (*t* = 0 to *t* = 30).

### 2.6. Statistical Analysis

Statistical analyses were performed using the statistical package SAS, version 9.2 (SAS Institute, Cary, NC, USA). Data are presented as mean ± SEM and *p*-values < 0.05 were considered statistically significant. Sample size was calculated based on our previous study [18]. We estimated that, given a coefficient of variation of 31.5%, an effect size of at least 15% between conditions, and a power of 0.80, we should at least obtain complete data from 35 subjects. To account for a drop-out rate of about 10% we aimed to enrol 40 subjects in the experiment. The data collected of subjects during a session in which they were not compliant to the MSF protocol was discarded and treated as missing data (see Section 2.1 and Figure 1).

First, energy intake and AUCs of satiety and well-being ratings of the four treatments were compared to the control condition by means of mixed model ANOVAs (PROC MIXED) with condition included in the model as fixed variable, and subject as random variable. *Post hoc*, a Dunnett test was used. Second, energy intake and AUCs of satiety and well-being ratings of the four treatments were investigated for separate effects of oral and gastric stimulation. This was done by mixed model ANOVAs (PROC MIXED) with oral stimulation duration (1 *vs.* 8 min), energy content (100 *vs.* 700 kcal) and their interaction term included in the model as fixed variables, and subject as random variable. *Post hoc*, if applicable, a Tukey test was used. Similar analyses were done to assess the effect for weight of the ingested food and for energy intake per meal-item, and to compare ratings at baseline and after the test meal. Baseline ratings were not included as co-variable since they did not differ between conditions.

## 3. Results

### 3.1. Energy Intake

**Figure 3:**
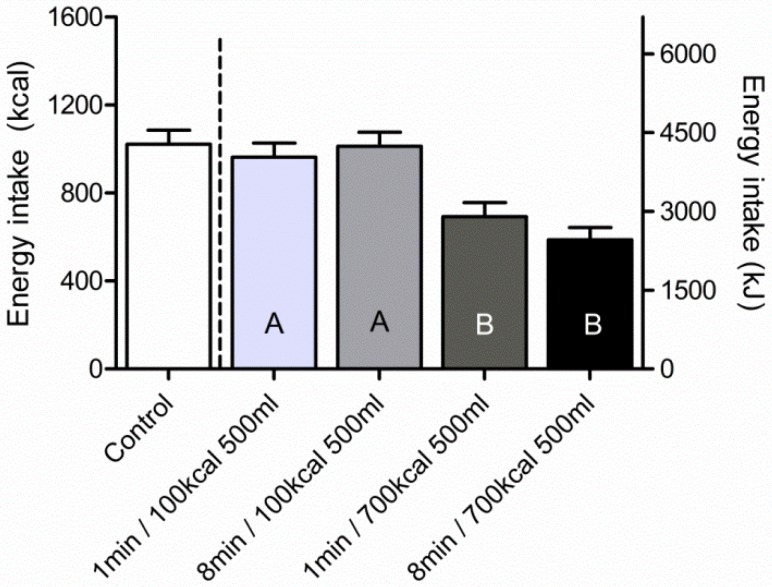
Energy intake per condition. Energy intake at the test meal is presented as mean kcal intake (± SEM). The test meal was offered half an hour after the treatment started and subjects could eat *ad libitum* for 30 min. Mixed model ANOVA on the 2 × 2 design showed significant effects of gastric load (*p* < 0.001) and gastric load × MSF (*p* < 0.03). Results of the *post hoc* Tukey tests are shown; different letters show statistically significant differences between treatments (*p* < 0.0001).

The mean energy intake for each condition is shown in Figure 3. Compared to control the 1 min/700 kcal and the 8 min/700 kcal treatment lowered energy intake significantly (Dunnett, *T* ≥ 7.44; *p* < 0.0001).

Energy intake was significantly lower after a high energy GLs compared to the low energy GLs (main effect of GL: *F* = 109.99; *p* < 0.0001). There was no main effect of oral exposure duration on energy intake (*F* = 0.84, *p* = 0.36). The ANOVA showed a significant interaction effect of GL and oral exposure duration (*F* = 5.07; *p* = 0.03), however *post hoc* analyses showed only statistically significant differences between the high energy and the low energy treatments, and no additional effects of oral exposure duration (see Figure 3).

### 3.2. Satiety Ratings

**Figure 4:**
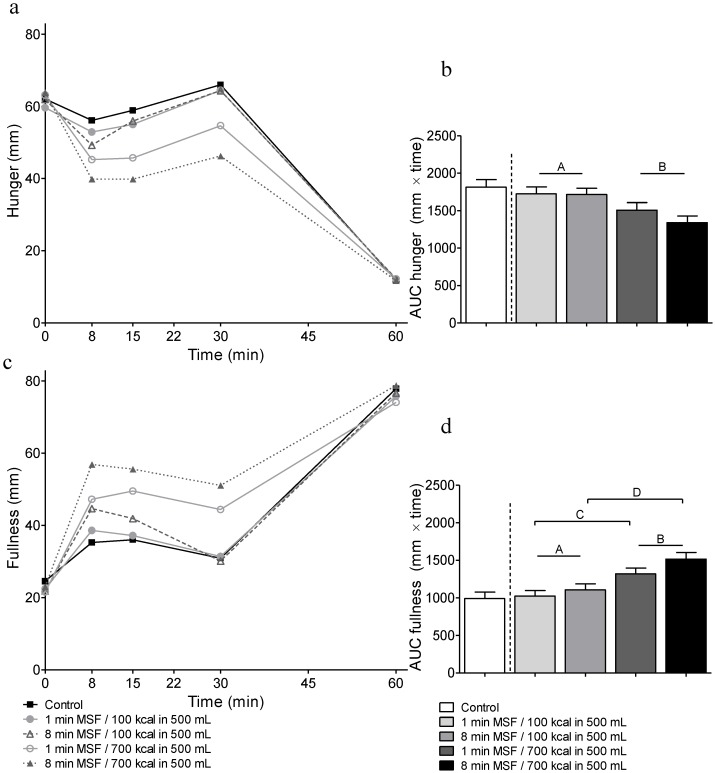
(**a**) Hunger ratings over time (**b**); areas under the curve (AUCs) of hunger ratings; (**c**) Fullness ratings over time; (**d**) AUCs of fullness ratings. Data are presented as means ± SEM. Hunger and fullness were rated on 100 mm visual analog scale lines and their AUCs were calculated from *t* = 0 to *t* = 30. Within the 2 by 2 design the 100 kcal gastric loads (GL) (A) resulted in more hunger and less fullness than the 700 kcal GLs (B) (effect of GL; *p*-values < 0.0001), and treatments with 1min modified sham feeding (MSF) (C) resulted in less fullness than treatments with 8 min MSF (D) (effect of MSF; *p* = 0.02).

Mean ratings of hunger and fullness over time and their AUCs are shown in Figure 4. Ratings of desire to eat, desire for sweet, desire for savory, and prospective consumption showed similar results as the ratings for hunger and are therefore not displayed. Satiety ratings after the test meal (*t* = 60) did not differ between conditions (*F* ≤ 2.24; *p* ≥ 0.07), indicating that subjects ate to the same level of satiation during every test meal.

An effect of energy content of the GL was found for all ratings: fullness increased and the other ratings decreased with higher gastric energy content (AUC; *F* ≥ 11.04; *p* ≤ 0.002). An effect of oral exposure duration was found for fullness and prospective consumption (AUC; *F* ≥ 5.88; *p* ≤ 0.02), but not for the other ratings (AUC; *F* ≤ 3.70; *p* ≥ 0.06). No effect for oral × gastric interaction was found (AUC; *F* ≤ 3.08; *p* ≥ 0.09).

### 3.3. Ratings of Thirst, Nausea, and Well-Being

Thirst, nausea, and well-being were not significantly different between conditions (AUC; *F* ≤ 1.93; *p* ≥ 0.11). In addition, no separate effects were found for oral and gastric stimulation (AUC; *F* ≤ 2.76; *p* ≥ 0.11).

## 4. Discussion

In the present study, we investigated the effect of -independently manipulated- oral exposure duration and gastric energy content on satiety ratings and energy intake. We found that a gastric load of 700 kcal led to higher satiety and lower energy intake in the next meal compared to an iso-volumetric gastric load of 100 kcal. We found no modifying effect of oral exposure duration—1 min MSF *vs.* 8 min—on energy intake in the next meal. Oral exposure duration by MFS affected some of the satiety ratings: larger AUCs for fullness and lower AUCs for prospective consumption were observed after 8 min MSF compared to 1 min MSF.

We observed a 35% (348 kcal) lower energy intake after the high energy GLs than after iso-volumetric low energy GLs. We made our difference larger than Rolls and Roe, who did not observe an effect as their difference between infusions was much smaller; 0.5 *vs.* 1.0 kcal/mL. In our former study, we showed the contrast in energy density—0.2 *vs.* 1.4 kcal/mL was large enough to observe a physiological difference, that is a difference in gastric emptying rate [19]. Besides energy content, the GL was also slightly different in viscosity. This might have been a confounding factor, however, the liquids were infused at the same rate, and the viscosity difference was only small as both liquids were easily infused by the nasogastric tube. Taking the findings of our studies together suggests that lower energy intake of the second meal occurred (partly) as a result of a pre-absorptive physiological effect, that is via a slower gastric empting of the stomach content in to the intestine.

Satiety ratings were also higher after the high energy loads and—but to a lesser extent—after the long oral exposure duration, *i.e.*, hunger was lower and fullness was higher after the oral exposure duration. Figure 4 shows that, in general, these effects were visible directly after the infusion, but that they only persisted for the treatments with a high energy GL. In other words, the effect of oral exposure duration on hunger and fullness is only short lived (<30 min) unless it is accompanied by a high energy dense gastric load. This finding is in line with our other study in which we investigated the effects of different volumes of a low caloric (100 kcal) gastric load [18] on appetite ratings; in this study the effects of a short or long oral sensory exposure on satiety were also short lived and disappeared after 30 min. In the other study [19], we also saw that oral exposure duration only had a small effect, which was very short lived, and that the effect of energy content persisted up to 90 min. These findings suggest that the effect of oral exposure duration is only important during and shortly after the meal and that later in time, gastric factors are more important.

The absence of a large effect of oral exposure on energy intake might be due to methodological limitations of our study. Although we have a very well controlled design, one might argue whether knowledge of the subjects might have influenced the results. We told them that the study was about a possible effect of chewing in combination with wearing a nasogastric tube on appetite. Although the subjects did not know the content of the gastric loads nor did they know that their food intake was measured, they were filling out appetite questionnaires and they knew that there was a difference in oral exposure duration. It has been shown that knowledge and merely thinking of food can already have an impact on certain physiological responses, such as gastro-intestinal motility, gastric secretion, and appetite measures [23,24]. The study of Cassady [23] illustrates this very well; subjects that were told that a liquid would become solid in their stomach showed slower gastric emptying rates and lower oro-cecal transit times, as well as higher satiety responses and lower test meal intake, compared to conditions in which they were told that the liquid remained liquid in their stomach . Endocrine responses, that is insulin, GLP-1 and ghrelin, were not as much affected by this information, and might be more difficult to influence by cognition.

In the treatments with the high energy GLs the cumulative energy intake—GLs plus test meal—was very high: *i.e.*, 1392 kcal in treatment 1 min/700 kcal and 1287 kcal in treatment 8 min/700 kcal. This corresponds to approximately 40%–50% of the daily energy needs of young healthy men. In the low energy treatments, this was much lower. Part of this large intake might be caused by the instruction that subjects had to stay seated for 30 min. In addition, subjects had no cues of the type or amount of food that was infused. It has also been shown previously by Wansink *et al.* that absence of visual cues of portion size can increase energy intake by 73% [25]. So it is likely that our high energy infusion presumably led to passive overeating. None of the subjects showed high responses on the side effects, which shows that the subjects were still pleasantly satiated.

In the current study, we choose sponge cake for the current study as this is easy to dissolve in a gastric load and convenient to use with the MSF paradigm. This sponge cake was high in fat and carbohydrates (Table 2); *i.e.*, 50% of the energy was derived from fat and 44% from carbohydrates. It has been shown that the nutrient composition of the food which is used for MSF affects cephalic phase responses [26,27]. For example, MSF of bananas (high carbohydrate) led to lower pancreatic polypeptide responses than codfish (high protein) or walnuts (high fat) [26]. Next, Zhu showed that MSF of nuts, cheese, and cereals also led to differential responses in insulin and ghrelin responses [27]. It might therefore very well be that our findings are only limited to the effects of oro-sensory exposure to sponge cake, and would be different if other foods were used.

Food intake is the result of a complex cascade of different psychological and physiological procesess [1]. Sensory and pre-absorptive signals affect satiation and satiety. Our study was not able to show an effect of oro-sensory duration on subsequent energy intake. This is not what we expected, considering the large number of studies that have shown that sensory cues are pre-absorptive signals that prepare the body for optimal digestion [2,28]. However, we also acknowledge that our experiment investigated included a very high energy dense gastric filling of 140 kcal/100 g. We speculate that any possible differences in cephalic phase responses might have been overshadowed by the strong gastric effects of the high energy dense gastric load. It might very well be that there would be difference in food intake if there was no oro-sensory exposure at all, like in the studies of Spetter [17] and Cecil [2] where a clear effect of oro-sensory exposure was found. The difference between no exposure and exposure might be more effective than the difference between 1 and 8 min of oral exposure. Adding a “no-oral exposure” condition is highly recommended in new studies. In addition, it might be that oro-sensory exposure has a small (but biologically relevant) effect, which is more relevant if gastric responses are smaller, like in our previous study were we had an energy density of 10 kcal/100 g [18].

Although our experiment was very well controlled, the current paradigm is far from normal eating. During normal food consumption, the digestion of the food already starts in the oral cavity upon contact with saliva, *i.e.*, amylases and lipases, start to break down starches and fat respectively. Next, foods are being swallowed and enter the stomach in boli and not in a continuous flow. Moreover, in our experiment, subjects spat out the sponge cake and wore a nasogastric tube. Although we only selected subjects that tolerated the nasogastric tube, we cannot discard effects of the nasogastric tube on appetite or physiological responses. Therefore, generalization of our observations to normal food consumption should be done with caution.

To conclude, our study shows that a gastric infusion of 700 kcal increases satiety and lowers subsequent food intake by 35% compared to an iso-volumetric gastric infusion of 100 kcal. An eight-fold longer oro-sensory exposure by means of MFS did not have an additional on food intake. Although we could not show the importance of oro-sensory exposure in the current experiment, with these manipulations, we still do think that oro-sensory exposure is important in food intake regulation as this has been shown by numerous other researchers.

For future research, we suggest not only investigating the effects on physiological markers of cephalic phase responses, such as insulin, ghrelin, and pancreatic polypeptide, but also to investigate the effect of different sensory stimuli and nutrients. In addition, it would be very interesting to investigate not only the duration of oro-sensory exposure, but also to add a condition with no oro-sensory exposure, as previous studies with either normal or non-sensory exposure have shown very convincing results [17,29]. It might be that simply having oro-sensory exposure, regardless the duration, already elicits a number of cephalic phase responses.

In a recent review of Mc**C**rickerd and Forde, it is postulated that in future research we should go beyond liking with sensory science and that sensory properties of foods have an important role in optimizing the satiating and rewarding value of foods [30]. Research in this direction will not only increase the fundamental understanding of oral stimulation in relation to satiety and food intake, but also the development of novel satiation foods which we think are essential in the scope of the current obesity epidemic and the high availability of fast eaten foods with only little sensory exposure.

## Supplementary Materials

Supplementary Materials are available online at www.mdpi.com/2072-6643/8/2/64/s1.
