# Supplementary material for: Effects of Oral Exposure Duration and Gastric Energy Content on Appetite Ratings and Energy Intake in Lean Men
Source: Nutrients. 2016 Jan 26;8(2):64. doi: 10.3390/nu8020064 (PMC4772028; doi:10.3390/nu8020064)
Supplement: Supplementary file 1 [file nutrients-08-00064-s001.docx]

Supplementary Materials: Effects of Oral Exposure Duration and Gastric Energy Content on Appetite Ratings and Energy Intake in Lean Men

Anne G. M. Wijlens, Cees de Graaf, Alfrun Erkner and Monica Mars

**Figure S1.** Hunger and fullness ratings of the control and treatment 1 min/800 mL of the present and previous study. This figure shows the mean ratings of hunger and fullness of the control condition and treatment 1 min/800 mL of the previous study on the left [[1](#_ENREF_1)] and of the present study on the right. Hunger and fullness were rated on 100 mm Visual analogue scale lines. The scales were anchored “not at all” on the left and “extremely” on the right.

**Figure S2.** Energy intake after the control and after treatment 1 min/800 mL of the present and previous study. This figure shows the energy intake (kcal ± SEM) at the test meal of the control and treatment 1 min/800 mL of the previous [[1](#_ENREF_1)] and present study. In both studies the test meal was offered half an hour after the treatment started and subjects could eat *ad libitum* for 30 min.

**Table S1.** Products and amounts offered at the test meal for the three energy groups.

|  | **2030–2746 kcal  per Day** | **2747–3462 kcal  per Day** | **3463–4080 kcal  per Day** |
| --- | --- | --- | --- |
| Bread rolls (pieces, each ± 28 g) | 15 | 20 | 25 |
| Margarine (g) | 150 | 150 | 150 |
| Cheese (g) | 80 | 120 | 160 |
| Chocolate sprinkles (g) | 150 | 150 | 150 |
| Ham (g) | 80 | 120 | 160 |
| Jam (g) | 200 | 200 | 200 |
| Water (g) | 150 | 150 | 150 |

**Table S2.** Energy content and macronutrient composition of test meal products ^a^.

|  | **Energy (kcal)** | **Protein (g)** | **Carbohydrate (g)** | **Fat (g)** | **Fiber (g)** |
| --- | --- | --- | --- | --- | --- |
| Bread rolls | 262 | 9.9 | 46.7 | 3.7 | 2.0 |
| Margarine | 697 | 0.0 | 0.0 | 79.0 | 0.0 |
| Cheese | 391 | 23.3 | 4.2 | 31.7 | 0.0 |
| Chocolate sprinkles | 501 | 6.0 | 68.3 | 22.6 | 7.5 |
| Ham | 123 | 13.3 | 5.6 | 5.3 | 0.5 |
| Jam | 255 | 0.2 | 62.6 | 0.0 | 0.8 |

^a^ Per 100 g, as determined by chemical analysis.

**Reference**

1 Wijlens, A.G.M.; Erkner, A.; Alexander, E.; Mars, M.; Smeets, P.A.; de Graaf, C. Effects of oral and gastric stimulation on appetite and energy intake. *Obesity* **2012**, *20*, 2226–2232, doi:10.1038/oby.2012.131.

© 2016 by the authors; licensee MDPI, Basel, Switzerland. This article is an open access article distributed under the terms and conditions of the Creative Commons by Attribution (CC-BY) license (http://creativecommons.org/licenses/by/4.0/).
